# Zonisamide for the Efficacy of Sleep Abnormality in Parkinson's Disease (ZEAL Study): A Protocol for Randomized Controlled Trials

**DOI:** 10.3389/fneur.2021.741307

**Published:** 2021-12-10

**Authors:** Hiroshi Kataoka, Masahiro Isogawa, Takashi Inoue, Miyoko Hasebe, Ryuzo Takashima, Shu Kasama, Hitoki Nanaura, Takao Kiriyama, Masato Kasahara, Kazuma Sugie

**Affiliations:** ^1^Department of Neurology, Nara Medical University, Kashihara, Japan; ^2^Institute for Clinical and Translational Science, Nara Medical University Hospital, Kashihara, Japan

**Keywords:** Parkinson, sleep, REM sleep behavioral disorders, clinical trial, zonisamide

## Abstract

**Background:** Sleep disorders are one of the most frequent non-motor symptoms of Parkinson's disease (PD), and the efficacy of dopaminergic agents remains controversial. Clinical randomized control trials for the treatment of sleep disorders in PD are limited. Zonisamide (1,2-benzisoxazole-3-methanesulfonamide) improved motor symptoms and wearing-off in patients with PD. Patients with PD were reported to have dream-enacting behavior that was resolved after treatment with zonisamide. This study aimed to verify the safety and efficacy of zonisamide for sleep disorders and rapid eye movement (REM) sleep behavioral disorders using a mobile two-channel electroencephalography (EEG)/electrooculography (EOG) recording system.

**Methods and Analysis:** The present study is a randomized placebo-controlled trial to determine the efficacy of zonisamide for sleep disorders in patients with PD. This study was designed to be single-blind, but the subject allocation is randomized by an independent allocation manager via computer-generated block randomization. The subjects in the treatment group took zonisamide (25 mg per day) before bedtime for 28 days. The sleep index is analyzed using a portable EEG/EOG recording system collected on two consecutive nights within 7 days prior to the intervention and reobtained on one night within 2 days after the 28-day administration of zonisamide. The amount of change in sleep efficiency before and after the 28-day administration will be compared between the zonisamide treatment group and placebo group concerning the primary endpoint. As for the secondary endpoint, the change in the ratio of other sleep parameters, including REM sleep without atonia, or sleep architecture will be evaluated.

**Ethics and Dissemination:** The protocol was approved by the Nara Medical University Certified Review Board (CRB5200002). The trial was notified and registered with the Japan Registry of Clinical Trials (jRCTs051200160). Written informed consent will be obtained from every participant using informed consent approved by the CRB. The results of this trial will be disseminated through peer-reviewed scientific journals.

## Introduction

Sleep disorders are one of the most frequent non-motor symptoms of Parkinson's disease (PD), and the efficacy of dopaminergic agents remains controversial (1, 2). PD-related sleep disorders can be influenced by primary neuronal loss in the dopaminergic, cholinergic, serotoninergic, or other systems that are intrinsic to PD or secondary effects such as dopaminergic effects or from sleep medications (1, 3). The multifactorial conditions include nocturnal motor symptoms, psychosis, hallucinations, urinary incontinence, depression, cognitive impairment, vivid dreaming, or rapid eye movement (REM) sleep behavioral disorders (RBD), which elicit sleep fragmentation and difficulties in maintaining or falling asleep. Sleep fragmentation is the most common sleep disorder in PD, with an estimated rate of 74–88% (4, 5). Sleep fragmentation is closely associated with many burdens on PD pathology (6). Vivid dreaming and its elicited sleep behavioral disorders, so-called “RBD” can produce nighttime awakening, leading to an increase in sleep fragmentation. Since idiopathic RBD is recognized as the prodromal stage of neurodegenerative diseases, especially in PD globally (7), and PD with RBD has a faster progression and more cognitive decline (8, 9), RBD has been increasing in importance. RBD is also related to the degeneration of the brainstem sleep regulatory center. Thus, the association between insomnia and RBD has intensified (10). Sleep disorders are an early indicator of PD (7). Sleep disorders and excessive daytime sleepiness or unsteadiness, with a potential risk of falling, independently affect the quality of life in patients with PD.

Benzodiazepines or non-benzodiazepine receptor agonists are widely used treatments for insomnia in non-PD populations; however, these can cause side effects such as drowsiness, cognitive decline, and daytime unsteadiness. Sleep contributes to dopamine receptor regulation and dopamine storage, resulting in pathophysiological progression in PD (6, 11, 12). Clinical trials for the treatment of sleep disorders in PD are limited. Long-acting rotigotine showed significant improvement in difficulty in staying asleep, nocturnal motor symptoms including akinesia, and frequency of nocturia in 287 patients (placebo in 97 patients) (13). Chronic-release levodopa/carbidopa improved nocturnal akinesia and increased the total sleep time in 40 patients including placebo in 15 patients (14). The benefit of prolonged-release melatonin on sleep quality was seen in another study including 34 patients who received placebo in 18 patients (15). A placebo-controlled crossover study of melatonin (5 vs. 50 mg) in 40 patients showed a significant improvement in total nighttime sleep on actigraphy during the 50-mg melatonin treatment (16). Conversely, the patient's reported total sleep time on their diaries did not increase in one study that evaluated the efficacy of eszopiclone in 30 patients with polysomnographic-measured sleep impairment (placebo in 15 patients) (17). Actigraphic sleep efficiency and sleep fragmentation worsened in one study of pergolide in 22 patients, including placebo in 12 patients (18). A study evaluating the effect of doxepin and the non-pharmacologic combined treatment of cognitive-behavioral therapy and bright light therapy in 18 patients (placebo in six patients) reported no change in sleep outcomes in both groups on actigraphy (19). No significant difference was observed in the polysomnographic measures between eight patients receiving melatonin and 10 placebo patients (20). Overall, some medications have a benefit in subjective sleep measurement; however, the efficacy of sleep parameters is unlikely to be vague.

Zonisamide (1,2-benzisoxazole-3-methanesulfonamide) was launched in Japan and was effective for the treatment of PD in clinical trials (21–23). It improved motor symptoms and wearing-off in patients with PD, with a very low incidence of dyskinesia and psychiatric symptoms such as hallucinations (21–23). Its pharmacological mechanisms remain unclear, and the effect of zonisamide on non-motor symptoms was uncertain since the clinical trial has not been studied. More recently, an open-label study showed the efficacy of treatment with zonisamide on PD-specific questionaries in 20 patients with PD receiving 25 mg (60%) and 50 mg (40%) (24). Zonisamide has a long-half life with a low potential for interacting with other medications (25) and its effect may improve the nocturnal motor symptoms, leading to subsequent improvement in sleep disorder. Moreover, in 2012, a patient with PD was reported with vivid nightmares and dream-enacting behavior that was dramatically resolved after treatment with zonisamide (26). In 2015, Ikeda et al. evaluated the therapeutic effects of zonisamide monotherapy in 10 patients with *de novo* PD, and three patients with a prior history of RBD markedly inhibited nocturnal symptoms of RBD after zonisamide treatment (27). These findings raised an open question as to whether zonisamide might have some potential improvement in sleep disorders in patients with PD.

This study aims to verify the safety and efficacy of zonisamide not only for sleep disorders but also for RBD using a mobile two-channel electroencephalography (EEG)/electrooculography (EOG) recording system.

## Methods and Analysis

### Participants and Recruitment

The present study is a randomized placebo-controlled trial to determine the efficacy of zonisamide for sleep disorders in patients with PD. All procedures were conducted at the Nara Medical University Hospital in Japan. A total of 70 patients were diagnosed with PD according to the International Parkinson and Movement Disorder Society (MDS) diagnostic criteria (28); however, the decline in the striatal uptake on the dopamine transporter in single-photon emission CT does not matter. All patients ≥41 years during the informed consent procurement have at least one sleep problem among the three of the following items: (1) they answered “Sometimes (2–3 times a week),” “Almost none (once a week),” or “Nothing” on item 1 scored regarding “Did you sleep well last week?”; (2) answered “Sometimes (2–3 times a week),” “Many (4–5 times a week),” or “So much (6–7 times a week)” on another item 2 scored regarding “Did you have a bad day at night?” on the PD Sleep Scale (PDSS)-2 Japanese version (29); or (3) they have a score ≥5 on the 10-item no/yes Sleep Behavior Disorder Screening questionnaire Japanese version (RBDSQ) with a maximum score of 13 (30). The PDSS-2 consists of 15 questions regarding PD-related nocturnal symptoms, and it is widely used as a recommended, valid, and highly reliable scale and the validity and reliability of the Japanese version were established (29). RBDSQ provides greater sensitivity and reasonable specificity for RBD compared with polysomnography (31). A cut-off score of ≥5 points has been found to have high sensitivity, specificity, and reliability in the Japanese population (29). All patients have a Mini-Mental State Examination score of ≥22 to preclude the following instructions. All patients are outpatients with Hoehn and Yahr stages 1 to 4 and received oral levodopa before entry. The regimen and dosage of antiparkinsonian medication were stable for at least 2 weeks prior to clinical trial participation.

The exclusion criteria includes the following: patients who had been treated with zonisamide within 3 months prior to obtaining informed consent, with a history of brain surgery including deep brain stimulation surgery, a history of other organic cerebral disorders such as stroke and epilepsy, hepatic or severe renal dysfunction with estimated glomerular filtration rate <30, taking antiepileptic drugs, taking both monoamine oxidase-B (MAO-B) inhibitors and tricyclic antidepressants, severe dyskinesia, severe mental disease, with potential and intention to become pregnant, with a history of malignant syndrome, with suicide attempts, toxic epidermal necrolysis, mucocutaneous ocular syndrome (Stevens–Johnson syndrome) or erythroderma (exfoliative dermatitis), history of hypersensitivity syndrome, interstitial pneumonia, or rhabdomyolysis within 5 years prior to obtaining informed consent, aplastic anemia, agranulocytosis, pure red cell aplasia, or thrombocytopenia, with pacemakers, and a history of hypersensitivity to the components. The subjects who were eligible according to the inclusion criteria provided written consent for participation in this trial.

### Randomization and Blinding

**Figure 1:**
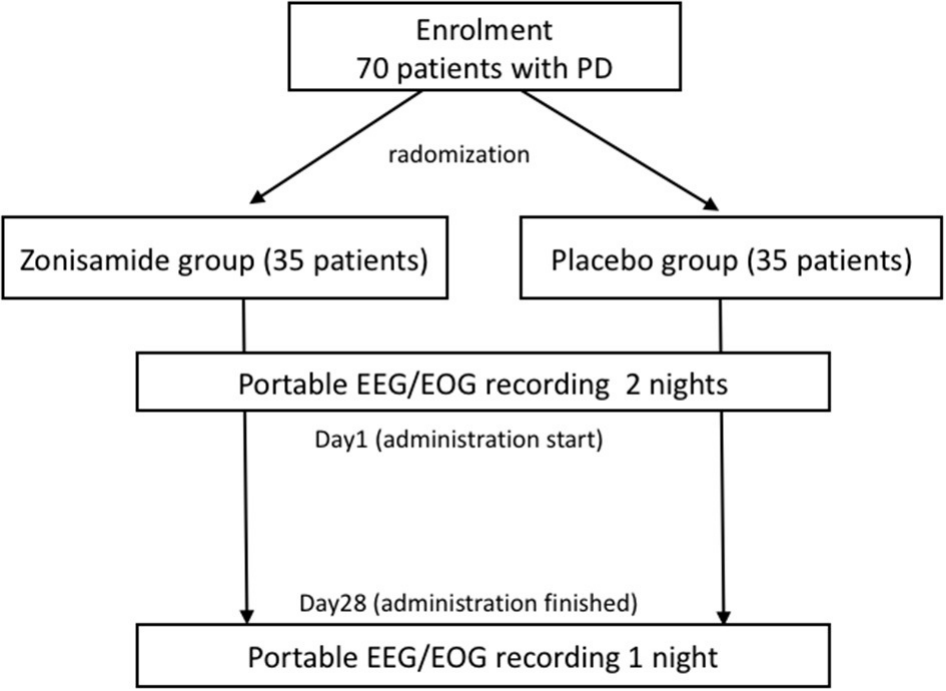
Flowchart of the study showing randomized control trials.

The patients from the treatment and control groups were randomized in a 1:1 ratio (Figure 1). This study is designed to be single-blind, but the subject allocation was randomized by an independent allocation manager *via* computer-generated block randomization.

### Intervention

The subjects in the treatment group took zonisamide (25 mg per day) before bedtime for 28 days. If the dosage of the following medications is stable from 2 weeks before enrollment to the initiation of the intervention, both groups are allowed to receive conventional anti-PD medications including L dioxyphenylalanine (L-DOPA), dopamine agonists, catechol-*O*-methyl transferase inhibitors, monoamine oxidase (MAO) inhibitors, and other antiparkinsonian drugs other than zonisamide, tricyclic or tetracyclic antidepressants, antiepileptic agents, reserpine derivative, phenothiazines, butyrophenone drug, sulpiride, or metoclopramide. All these medications were prohibited from new dosing during the clinical trial period, and a constant dose of these medications was maintained during the intervention. The conventional treatment for comorbidity and complications, sleep stabilizers, or other medications related to PD, such as antidementia drugs, antipsychotics, anxiolytics, or selective serotonin uptake inhibitors, will be allowed, but these dosages will be stable during the clinical trial. Supportive care for maintaining PD status such as rehabilitation before the clinical trial participation's consent would be performed; however, surgery such as deep brain stimulation would not be tolerated. The criteria for discontinuing this clinical trial are withdrawal of the trial participation agreement by subjects or their legal representatives, violation of the selection/exclusion criteria, the occurrence of adverse events that make it difficult to continue the clinical trial, and <70% compliance with the medication criteria.

The schedule of the intervention and outcome measurements is shown in Figure 1.

To improve adherence to clinical trials, this clinical trial will be regulated to monitor patient safety, adverse effects, quality control of the data, eligibility of patients, and protocol violations by independent monitoring committees and clinical research associates. Moreover, it will be audited thrice during the trial by a professional company unrelated to the research teams. Safety assessment was performed for all subjects through clinical symptoms and features, which were conducted by telephone interview and clinical examination during a visit to the hospital, and was performed thrice at baseline before drug administration, 14 days after drug administration, and during drug administration. When the patients note undesirable and/or unexpected medical findings, which did not occur prior to the clinical trial, it is defined as an adverse effect; the subject will consult the doctor of the trial committee, and the event will be recorded at any time during the course of the trial. The protocol for this trial was approved by the research ethics committee of the Nara Medical University and was registered with the Japan Registry of Clinical Trials (jRCTs051200160).

### Primary Outcome

The primary outcome was sleep efficiency (SE), which is the percentage of actual sleep time during sleep (%).

### Secondary Outcomes

The secondary outcomes are listed as follows:

Objective outcome

1. Total sleep time (TST): time from falling asleep to final awakening (min).
2. Wake time after sleep onset (WASO): total awakening time during sleep (min)
3. Sleep onset latency (SOL): time from bedtime to the initiation of sleep (min).
4. REM sleep/non-REM sleep ratio (%).
5. Deep sleep (N3) time (minutes).
6. Automatically calculated ratio of REM sleep without atonia (RWA) to total REM sleep epochs (%)

Subjective outcome

1. PDSS-2
2. Pittsburg Sleep Questionnaire
3. RBDSQ

**Figure 2:**
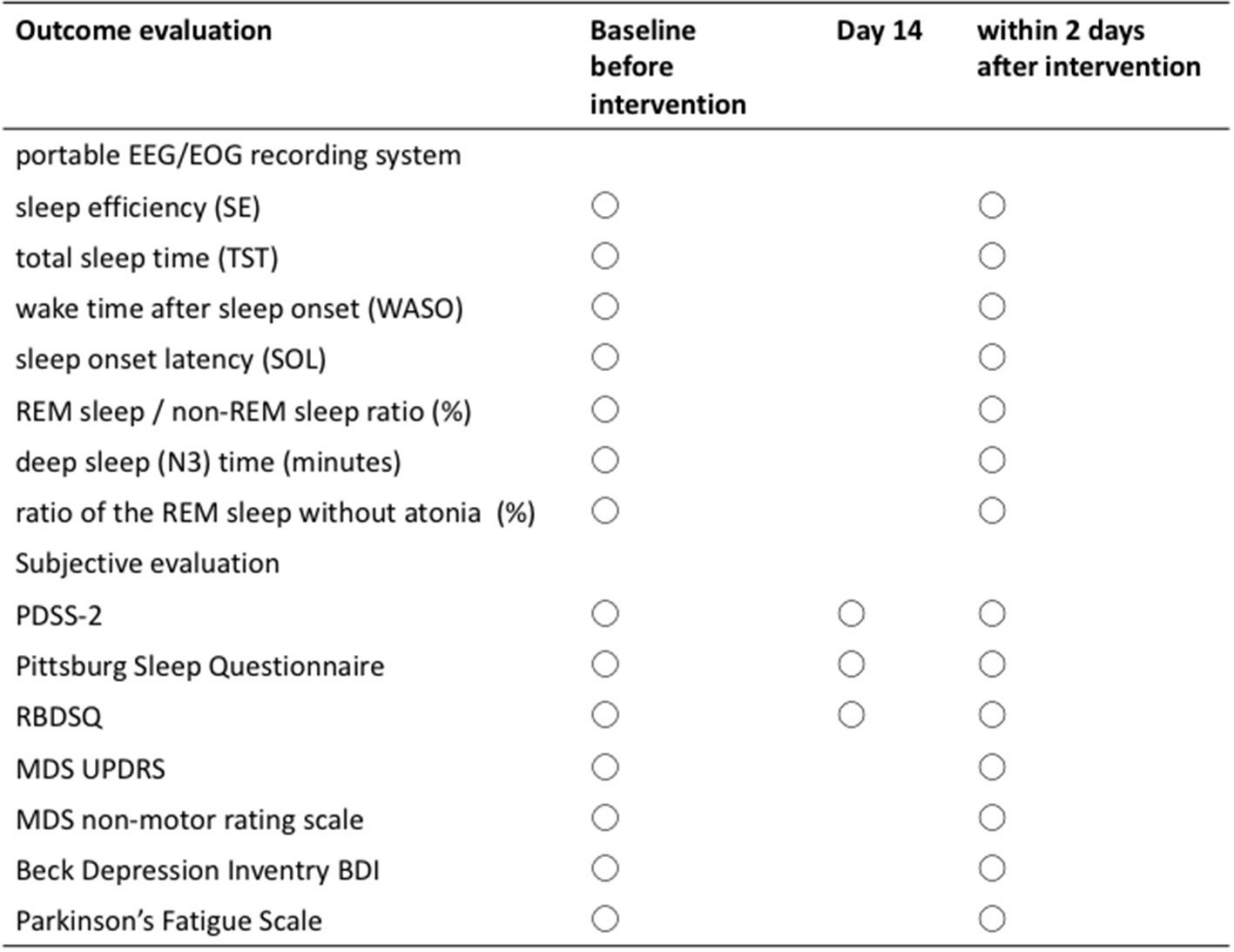
Schedule of examinations and procedures.

The sleep index is analyzed using a portable EEG/EOG recording system collected on two consecutive nights within 7 days prior to the intervention and reobtained on one night within 2 days after the 28-day administration of zonisamide, as shown in Figure 2. PDSS-2, Pittsburg sleep questionnaire, and RBDSQ were collected at baseline, 14 days after the administration and 2 days after the 28-day administration. Other clinical evaluation tools such as the Movement Disorder Society (MDS) Unified Parkinson's Disease Rating Scale part 3 and part 4 (32), item “psychosis” and “anxiety” on MDS non-motor rating scale (33), Beck Depression Inventory second edition (34), and Parkinson's Fatigue Scale (35) were performed.

*Portable EEG/EOG recording system* (SleepGraph; Medical Device Certification Number: 231AHBZX00001000).

A portable recording system (ZA) (SleepGraph®, Proassist Co., Japan) comprises a wire pair of bipolar EEG and EOG electrode leads, and the receiver was used for recording the frontal EEG and EOG (36). The forehead EEG was recorded from Fp1 with the opposite mastoid process (M2) as a reference, and the EOG electrode records not only electromyography (EMG) (high-frequency waves) but also EOG (low-frequency waves) from the two electrodes on the opposite chin muscle and the skin about 1 cm below the eyes. The signals are recorded at a sampling rate of 128 Hz with filters of 0.5–40 Hz for EEG and of 0.5–44 Hz for EOG. The amplified and filtered analog data from the electrodes were converted into digital signals using a 14-bit A/D converter, transmitted to the receiver placed on the bedside, and stored for offline data analysis. Sleep stage scoring based on the forehead EEG signals was shown in the literature (36), and its following sleep measurements as SE, TST, WASO, and SOL in addition to sleep stage structure were calculated according to the AASM rules. When the duration of phasic muscle activity lasts 0.1–5 s, and the amplitude is four times more than that of the background; EMG activity would be occupied more than 50% on the mini-epoch for 3 s. The epoch is defined as RWA, and the ratio of the RWA to total REM sleep epochs is automatically calculated. The portable EEG/EOG recording system, which objectively assesses sleep at home, is a self-applicable and affordable method. The estimated sleep variables were well-correlated between the portable EEG/EOG recording system and polysomnography, and the interscorer reliability for the sleep stage scoring and sleep variables between them was similar in healthy adults in the validation study (37).

### Statistical Analysis

The full analysis set (FAS) is defined as the population excluding any of the following: not meeting the eligibility criteria, having received zonisamide within 3 months prior to the trial patient's consent, or having no data after allocation. The per-protocol set is defined as the population excluding participants who violated a protocol from the FAS. The safety analysis set (SAS) is defined as the population receiving zonisamide. The amount of change in SE after the 28-day administration will be compared between the zonisamide treatment group and the placebo group in FAS regarding the primary endpoint. For the secondary endpoint, the change in the ratio of the RWA to total REM sleep epochs, TST, WASO, SOL, the ratio of REM/non-REM sleep, and deep sleep time or alteration of each score of PDSS-2, Pittsburg sleep questionnaire, or RBDSQ will be statistically compared between the treatment group and placebo group in FAS. Adverse event rates are evaluated between the two groups in SAS. The analysis methods include Student's *t*-test, Welch's test (for normally distributed patients with equal or unequal variances, respectively), or Wilcoxon's rank-sum test (for non-normally distributed patients). Statistical significance is defined as a two-sided *p*-value <0.05.

## Discussion

Causal variables for sleep disorders in PD are multifactorial, such as nocturnal motor symptoms, psychosis, depression, and RBD. Sleep parameters obtained from objective gold-standard polysomnography are disturbed in patients with PD rather than in healthy subjects, and these persist during the disease course, and some parameters such as sleep fragmentation, including WASO or RBD, become worse according to the severity of PD (38). Self-report sleep diaries or questionnaires have been widely used in studies, including clinical trials. Each instrument has its own strengths and limitations. The portable EEG/EOG recording system can evaluate natural sleep in the setting of the home, and its advantage of a portable EEG/EOG recording system makes it possible to objectively assess not only conventional sleep parameters but also RBD. The wireless single-channel headband sleep system had moderate-to-high sleep staging agreement between this system and polysomnography in healthy subjects (39); however, there has not been a study validating subjects with the disease. The portable EEG/EOG recording system can objectively diagnose RBD in an outpatient care setting. The present clinical trial evaluated the efficacy of the portable EEG/EOG recording system for the first time.

The mechanism by which zonisamide improves sleep quality is uncertain, but its administration before bedtime might ameliorate nocturnal motor symptoms. Dopaminergic dysfunction may play a role in the pathophysiology of RBD (40). Dopamine modulates the expression of locomotion and other rhythmic motor patterns in central pattern generators (41). The brainstem locomotor pattern generator is responsible for RBD is thought to be modulated by the zonisamide-modified dopaminergic system.

The limitation of this study is the low dose zonisamide administered (25 mg/day). Zonisamide 50 mg/day has revealed a decline in the severity of wearing-off, and its observation can further improve nocturnal motor symptoms.

## Ethics Statement

The studies involving human participants were reviewed and approved by Nara Medical University Certified Review Board (CRB5200002), Japan Registry of Clinical Trials (jRCTs051200160). The patients/participants provided their written informed consent to participate in this study.

## Author Contributions

HK, MI, TI, MH, RT, HN, TK, MK, and KS were responsible for the overall study design. HK wrote the manuscript. HK, MI, TI, SK, MK, and KS contributed to the drafting and critical revision of part of the submitted materials. All authors contributed to the article and approved the submitted version.

## Funding

The authors declare that this study was funded by Sumitomo Dainippon Pharma Co., Ltd. The funder was not involved in the study design, collection, analysis, interpretation of data, the writing of this article or the decision to submit it for publication.

## Conflict of Interest

The authors declare that the research was conducted in the absence of any commercial or financial relationships that could be construed as a potential conflict of interest.

## Publisher's Note

All claims expressed in this article are solely those of the authors and do not necessarily represent those of their affiliated organizations, or those of the publisher, the editors and the reviewers. Any product that may be evaluated in this article, or claim that may be made by its manufacturer, is not guaranteed or endorsed by the publisher.
